# Transversely Excited Multipass Photoacoustic Cell Using Electromechanical Film as Microphone

**DOI:** 10.3390/s100605294

**Published:** 2010-05-26

**Authors:** Jaakko Saarela, Johan Sand, Tapio Sorvajärvi, Albert Manninen, Juha Toivonen

**Affiliations:** 1 Optics Laboratory, Department of Physics, Tampere University of Technology, P.O. Box 692, FI-33101 Tampere, Finland; E-Mails: johan.sand@tut.fi (J.S.); tapio.sorvajarvi@tut.fi (T.S.); juha.toivonen@tut.fi (J.T.); 2 Empa, Swiss Federal Laboratories for Materials Science and Technology, Laboratory for Air Pollution & Environmental Technology, Überlandstr. 129, 8600 Dübendorf, Switzerland; E-Mail: Albert.Manninen@empa.ch

**Keywords:** multipass, photoacoustic, spectroscopy, longitudinal resonance, transversal excitation, transducer, electromechanical film microphone, EMFIT film, 82.80.Kq, 07.88.+y, 07.07.Df, *43.20.Ks, 43.38.Kb, 77.55.H-

## Abstract

A novel multipass photoacoustic cell with five stacked electromechanical films as a microphone has been constructed, tested and characterized. The photoacoustic cell is an open rectangular structure with two steel plates facing each other. The longitudinal acoustic resonances are excited transversely in an optical multipass configuration. A detection limit of 22 ppb (10^−9^) was achieved for flowing NO_2_ in N_2_ at normal pressure by using the maximum of 70 laser beams between the resonator plates. The corresponding minimum detectable absorption and the normalized noise-equivalent absorption coefficients were 2.2 × 10^−7^ cm^−1^ and 3.2 × 10^−9^ cm^−1^WHz^−1/2^, respectively.

## 1. Introduction

Photoacoustic spectroscopy (PAS) is a sensitive technique for trace gas analysis. The photoacoustic (PA) technique is based on the detection of sound waves that are generated due to the absorption of modulated light. The acoustic wave is usually excited inside a closed cell, having larger dimensions than the wavelength of the acoustic wave. When the frequency of the acoustic wave is matched to the resonance frequency of the PA cell, the acoustic wave is amplified constructively by the quality (Q) factor of the resonance. The sound is detected with a transducer, such as a condenser, or an electret microphone [1]. Optical microphones [2] and quartz tuning forks [3] have also been used. Recently, an electromechanical film was also applied for PAS of gases [4].

The electromechanical film (EMFi or EMFIT film) is a flexible, approximately 70 *μ*m thick, cellular polypropylene film with an internal charge [5]. The film has thin metal electrodes on its both sides. When a dynamic force is applied onto the film, opposite electric charges are generated on the two electrodes, from which the electric signal is measured. A single film layer has a wide frequency bandwidth with a flat response, even up to 300 kHz where the electromechanical resonance occurs. Below its resonance frequency the film has a sensitivity of about 2 mVPa^−1^. Furthermore, the films can be stacked to improve the sensitivity [6]. Due to the increased inertia of a film stack the resonance frequency reduces to tens of kilohertz [5,7] which, however, is usually above the operation frequencies of PA detectors.

Commonly, closed cylindrical PA cells are used together with radial, azimuthal, or longitudinal resonances. The Q values of cylindrical resonators are typically in the range of 10–1,000. Spherical resonators with even higher Q values have also been used. In cylindrical PA cells the radial resonances have their pressure maxima on the cylindrical axis. The optimum excitation of the radial resonances is achieved by aligning a small-diameter laser beam along the cylindrical axis [1].

In addition to the acoustic resonances, the PA signal can also be enhanced by optical multipass- or cavity-enhanced arrangements [8–17]. The increase of the sensitivity of a multipass PA system is based on the increased light absorption path length when the light beam is reflected multiple times through the sample volume. In intracavity systems the PA cell is placed inside a laser resonator [8–10]. On the other hand, in extracavity PA systems resonant optical cavities are seldom used because they need to be stabilized continuously and actively in order to maintain the optical resonance of the cavity. Instead, non-resonant multipass cavities are the most common.

In a Herriott-type multipass cavity the laser beam is introduced off-axially into a cavity, made of two mirrors, with the PA cell placed in between, so that the reflection of the beam occurs at a different spot after each roundtrip. The closer the beam family is to an antinode of a radial resonance of the PA cell, the better is the coupling of light energy into acoustic energy. However, due to the reflection losses of the mirrors, the PA signal enhancement is not directly proportional to number of roundtrips [11–13].

Optical multipass-enhancement of longitudinal acoustic modes has also been exploited. The laser beam can be introduced through a small aperture into a cylindrical PA cell, perpendicularly or transversally to the symmetry axis [14]. In this approach, the laser beam reflects multiple times from the inner metal walls of the PA cell until it is attenuated or exits through the entrance hole. Similar transversal illumination has been used in a Helmholtz resonator [15]. Absorbtion of light at the cell walls induces an inherent PA background signal, especially if amplitude modulation is used instead of wavelength modulation.

The PA background signal and the reflection losses can be minimized simultaneously by using a windowless multipass PA cell where the laser beam is reflected between high-reflectivity mirrors that are outside the open PA cell. This kind of open configuration was used with a Herriott-type PA cell [11] and recently with a nonresonant PA cell that was aligned between two planar mirrors [18].

In this work, a 5-layer EMFIT film is applied as a transducer in a novel PA cell where both acoustic longitudinal resonances and optical multipasses are employed to enhance the sensitivity of the PA detector. The PA resonator is a simple rectangular structure with two steel plates, facing each other, and with open sides. The acoustic wave, which resonates between the plates, is excited transversally in an optical multipass configuration where the reflected laser beams lie in a plane, parallel to the plates. The optical multipass configuration, used in this research, was adopted from the previous study [18]. 308 ppm (10^−6^) of nitrogen dioxide (NO_2_) diluted in pure nitrogen (N_2_) was used as a sample gas. The PA signal was excited within the visible rovibronic absorption band of NO_2_, using amplitude-modulated blue laser light. The absorption cross-section of NO_2_ at the 473 nm wavelength used was 4 × 10^−19^ cm^2^ [19].

The sensitivity of the longitudinally resonant PA detector is compared with a cylindrical PA resonator that was used in a previous study [4]. It is demonstrated that the open longitudinal and the closed radial PA resonators give comparable sensitivities in a single pass measurement if they have the same Q values and similar EMFIT microphones. Additional PA signal improvement is achieved by multipassing the laser beam inside the transversally excited PA resonator. With a maximum of 70 laser beam passes a detection limit of 22 ppb of NO_2_ in N_2_ was achieved. This corresponds to a normalized noise-equivalent absorption coefficient (NNEA) of 3.2 × 10^−9^ cm^−1^WHz^−1/2^ which is close to the best values that have been measured with PA methods from a flowing gas sample. Future improvements of the PA detection of trace gases, using EMFIT film microphones, are discussed.

## 2. Experimental

### 2.1. Photoacoustic instrumentation and measurements

**Figure 1.**
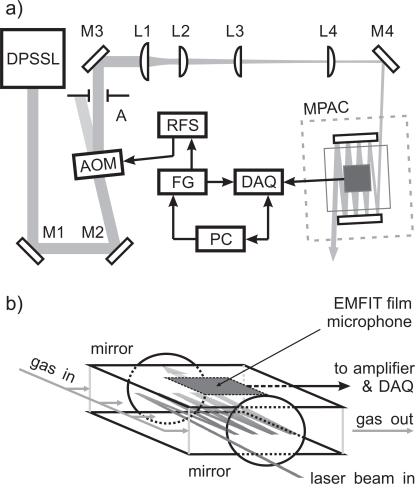
(a) Measurement setup: diode-pumped solid state laser (DPSSL), multipass photoacoustic cell (MPAC), function generator (FG), radio frequency power source (RFS), acusto-optic modulator (AOM), data acquisition (DAQ), personal computer (PC), mirrors (M1–M4), aperture (A), cylindrical lenses (L1–L2), and spherical lenses (L3–L4). (b) Multipass photoacoustic cell (MPAC). The reflected laser beam generates an acoustic plane wave source and excites longitudinal acoustic resonances.

The schematic of the PA measurement set-up is shown in Figure 1a. A novel resonant photoacoustic cell with an optical multipass configuration was used in this work. The longitudinal PA resonator had rectangular steel plates (10 cm × 10 cm), facing each other 2.4 cm apart as is illustrated in Figure 1b. Five EMFIT films (Emfit Ltd., Vaajakoski, Finland), each of them having a size of 4 cm × 4 cm, were stacked together with a thin layer of epoxy-glue. The internal polarizations of the films were aligned parallel to each other so that the overall voltage across the capacitive film stack was the sum of the voltages of individual films. The film stack is an equivalent to capacitors, connected in series. Afterwards, the stack was glued onto the upper steel plate with electrically conducting epoxy so that the back plate served as one of the metal electrodes. Electrical connections from the steel plate and the front surface of the film stack were wired into an input of a self-made voltage amplifier, which had a 60 dB amplification.

With the EMFIT film stack facing into the PA resonator, the two rectangular plates were separated with hollow 24-mm-long aluminum retainer tubes and tightened together with screws. Two broadband dielectric mirrors (ø = 50.8 mm, BB2-E02, Thorlabs) were placed on the opposite sides of PA resonator, forming an optical multipass cavity. The multipass PA cell (MPAC) was enclosed inside an aluminum box. The enclosure was equipped with windows as input and output ports for laser light. The NO_2_ sample gas (308 ppm of NO_2_ in N_2_, AGA, Finland) was introduced into the sample volume of the MPAC through a teflon tubing whose terminal end was drilled with a row of small holes in order to guarantee a uniform input flow of the sample gas. The measured concentrations between 1–30 ppm were diluted from the certified 308 ppm NO_2_–N_2_ gas mixture with a cascade of two mass flow controllers (5850S, Brooks Instrument, Hatfield, PA, United States), whereas the concentrations of 30–308 ppm were generated with only one of the controllers. In order to maintain a stable gas flow through the cascade, a pressure relief valve was used between the two controllers.

An acusto-optic modulator (MT110-A1.5-VIS, AA Opto-Electronic, Orsay, France) was used to amplitude-modulate the laser beam that was emitted from a diode-pumped solid state laser (Cobolt Blues™, Cobolt AB, Stockholm, Sweden), having an emission power and wavelength of 40 mW and 473 nm, respectively. A radio-frequency power source (MODA110-B4-33, AA Opto-Electronic) provided a 110 MHz carrier wave to the acusto-optic modulator (AOM). The carrier wave was square-wave modulated with a programmable function generator (33250A, Agilent Technologies, Santa Clara, CA, United States) via a LabVIEW software. As a result, approximately 27 mW of optical peak power was achieved into the first diffraction order of the AOM. The modulation frequency of the output of the AOM can be tuned up to 32 MHz. However, in PA measurements only modulation frequencies of 0.1–60 kHz were used due to a limited frequency response of the microphone–amplifier circuit.

The amplitude-modulated laser beam was guided and focussed into the MPAC. In Figure 1 a L3 and L4 are spherical plano-convex lenses with focal lengths of 30 cm and 20 cm, which are used as a lens pair to collimate the slightly diverging laser beam. The lenses L1 and L2 are cylindrical plano-convex lenses, having 10 cm and 3 cm focal lengths. They had an effective focal length of 1.2 m, and they focussed the laser beam horizontally into the PA resonator, bypassing the first mirror of the MPAC. The size of the beam [full width at half maximum (FWHM)] at the entrance mirror was 0.2 mm × 0.8 mm in the horizontal and vertical directions. The average laser power at the input of the MPAC was approximately 10 mW. The laser power was measured with a power meter (PD300-UV, Ophir Optronics Ltd., Jerusalem, Israel).

Due to the light absorption by NO_2_ molecules, a pressure wave is generated at the modulation frequency of the plane of laser beams. The PA signal is measured as the output voltage of the amplifier with a high-speed digitizer (NI USB-5133, National Instruments, Austin, TX, United States), DAQ. The data acquisition is controlled and the measurement data is stored to a personal computer (PC) with same LabVIEW software that controls the function generator (FG). In the PA measurements typical data sampling frequencies (*f_s_*) and data record lengths (*T_m_*) were 250 kHz and about 2.1 s, respectively. Their product corresponds to a total of 2^19^ data points. A fast Fourier transform (FFT) was calculated from the collected PA signal data. In a FFT spectrum the even number of 2*N* data points, where *N* is a positive integer, are presented at frequencies *f_k_* = *k* × Δ*f* = *k/T_m_*, where *k* = −*N*, ..., *N* − 1 [20]. When measuring the PA response of the MPAC as a function of frequency, the modulation frequencies of the laser beam were selected in such a way that they coincided with the frequencies *f_k_* ≥ 0, which were predetermined by *f_s_* and *T_m_*. The FFT spectrum was calculated after each PA measurement, using Hanning windowing, and only the root mean square (RMS) voltage at the modulation frequency is stored into the PC’s hard disc for further data analysis.

### 2.2. Microphone response measurements

The sensitivity and the frequency response of the 5-layer EMFIT microphone was compared with a calibrated reference microphone (BK Type 4939, Brüel&Kjær Sound&Vibration Measurement A/S, Nærum, Denmark), which had a measurement bandwidth of 0–100 kHz. The signal from the reference microphone was first connected to a preamplifier (2670, Brüel&Kjær) and then to a programmable amplifier (SR650, Stanford Research System Inc., Sunnyvale, CA, United States) having a high-pass cut-off frequency and a gain factor of 100 Hz and 60 dB, respectively. The response of the 5-layer film was also compared to that of a single film. A single EMFIT layer is known to exhibit response to sound waves up to 300 kHz. The operation of traditional loudspeakers that are used as calibration sound sources, are usually limited to audio frequencies.

The free-field responses of the three microphones were measured by PA means. Laser pulses from a Q-switched OPO laser system (NT342/1/UVE, Ekspla Ltd., Vilnius, Lithuania) were guided onto a solid steel sample at about 40° incidence. The pulse energy, duration, wavelength, and the diameter of the impact area were 60 mJ, 5 ns, 355 nm, and 5 mm, respectively. The high-intensity laser pulses heat the metal surface. A part of the heat is released to the surrounding air and, as a result, an acoustic pulse is emitted.

The acoustic pulse was measured with the three microphones, which were separated by 6 cm, at a distance of 1.8 m from the source. Thus, the microphones measured the acoustic far-field of the source roughly from the same solid angle. A thousand microphone signals were recorded and averaged with an oscilloscope (Waverunner 6100A, LeCroy Co., Chestnut Ridge, NY, United States). In order to resolve acoustic frequencies of several hundreds of kHz, a sampling frequency of 2.5 MHz was used. The frequency responses were achieved by calculating FFT spectra from the measured data.

## 3. Results and Discussion

### 3.1. Microphone response

**Figure 2.**
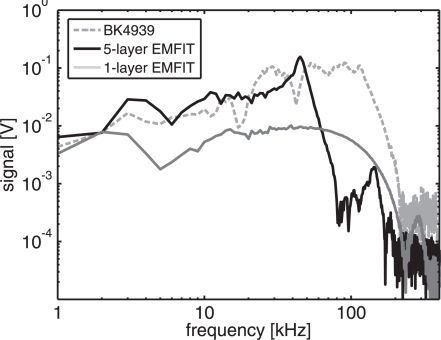
Photoacoustic response of a stack of 5-layer EMFIT film microphone in free field, compared with the responses of a 1-layer film and a reference condenser microphone. Due to the layering of the microphone films, the electromechanical resonance of the 5-layer microphone is reduced to 45 kHz from 280 kHz of the single microphone film. The layering enhances the microphone signal by a factor of 4–5 at low frequencies.

The acoustic response of the 5-layer EMFIT film microphone, used as the transducer in the PA measurements of NO_2_, is compared to that of a commercial condenser microphone (BK Type 4939) in Figure 2. The response of a single film is also presented. The responses were measured by PA means as described in Subsection 2.2. It can be seen that the single EMFIT film has its electromechanical resonance at 280 kHz, whereas the resonance frequency of the 5-layer EMFIT stack is around 45 kHz. Below this frequency the film stack has approximately 4–5-fold sensitivity when compared to the sensitivity of the single film. Furthermore, the layered films give a signal that is comparable with that of the reference microphone whose reported sensitivity is 4.14 mV/Pa at 250 Hz.

The measured amplitude spectra of Figure 2 could be presented as absolute sensitivity curves by dividing them with the frequency distribution of the incident pressure wave. This, however, was not done because there are several local minima and maxima in the reference spectrum below 100 kHz which are not seen in the smoother spectra of the EMFIT films. The existence of these features is probably related to acoustic interference. Different frequency components exhibit different directionality patterns when the diameter of the source is comparable to the wavelength of sound [21], as now is the case at ultrasonic frequencies. Thus, the transducers are affected by the directionality and interference. Due to the large size of the EMFIT films, the interference is averaged out, and smoother spectra are measured than with the 1/4″ reference microphone.

### 3.2. Performance of planar photoacoustic cell

The performance of the novel MPAC was compared with a cylindrical PA cell that was used in the previous study [4]. The cylindrical PA cell was optimized for the excitation of radial eigenmodes and for optical single pass measurement. The cylinder had a length and radius of 10 cm and 1.85 cm, respectively, and half of the inner surface was covered with a folded 2-layer EMFIT film. The 2nd radial resonance of the cell was at 21.06 kHz. In order to compare the performances of the planar MPAC and the cylindrical cell, the plate distance of the MPAC was adjusted to 1.6 cm to give a resonance frequency of 22.62 kHz for the 2nd longitudinal mode. In addition, the 10 cm × 10 cm MPAC resonator was equipped with an EMFIT film of similar size and sensitivity as used in the cylindrical PA cell.

A cylindrical laser beam, having a diameter of 0.8 mm (FWHM), was aligned collinearly along the cylindrical axis of the PA cell. The spherical beam profile that is commonly achieved from laser sources, is ideal for the excitation of radial acoustic modes, whereas a wide rectangular profile would be the optimum beam shape for the planar longitudinal waves. A wide elliptical beam profile serves as a close approximation to the rectangular profile. Therefore, a short focal length cylindrical lens was used to divert the spherical, gaussian laser beam into an elliptical profile, having a minor and major axes of about 0.8 mm and 20 mm, respectively.

The initial pressure wave amplitudes of the two different PA cell types can be compared by normalizing their PA signals with the corresponding laser powers and Q values. The Q values of the 2nd order modes of the cylindrical and planar PA cells were about 530 and 220, respectively. The Q value was defined as a ratio of the resonance frequency and the width of the resonance profile at 2^−1/2^ height. Consequently, a difference of a factor of two in the initial amplitudes was found for the benefit of the cylindrical PA cell. This probably results from the more efficient PA coupling between the spherical laser beam and the radial mode, compared to the coupling between the elliptical beam and the longitudinal mode. Nevertheless, with a single beam pass the sensitivity of the novel transversely excited planar PA cell was about 5 times lower than the sensitivity of the similar cylindrical PA cell with axial excitation.

### 3.3. Multipass photoacoustic measurements

**Figure 3.**
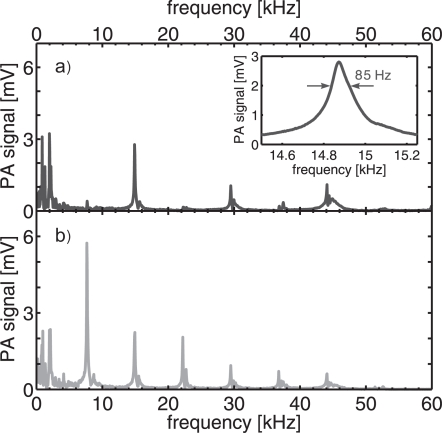
Acoustic resonances of the planar MPAC with the plane of multipassed laser beams lying horizontally (a) in the center and (b) at the bottom of the cell. The longitudinal resonances occur above 7.86 kHz. The 2nd longitudinal resonance at 14.87 kHz, having a Q value of 175, is shown in the inset. Only even longitudinal modes are excited when the laser beams are located in the center of PA cell.

The frequency response of the PA detector is presented in Figure 3. The 5-layer film microphone and its mounting plate were attached to the MPAC as the top plate of the resonator, and the plate separation was adjusted back to 2.4 cm. A plane of 21 laser beams was aligned parallel to the resonator plates. When the laser beams are located on the bottom of the PA resonator, all the longitudinal acoustic resonances can be excited. The fundamental longitudinal resonance occurs at 7.86 kHz, and the higher harmonics occur approximately at its multiples. On the other hand, when the beams are aligned along the central plane of symmetry only even harmonics can be excited. This is due to the fact that only even harmonics have an antinode in center of the resonator. Then, the induced pressure wave is amplified constructively as it oscillates between the resonator plates. Furthermore, the amplitudes of the even harmonics are maximized with central excitation because the up- and downwards propagating pressure waves, which are generated by the plane of laser beams, oscillate in phase with each other. The large scale spectra in Figure 3 a and b were measured with a resolution of 50 Hz. The spectrum in the inset, where the profile of the 2nd longitudinal eigenmode is shown, was measured with a resolution of 5 Hz. The Q value is 175 for the the PA cell with the 2.4 cm plate distance.

By measuring the peak value of the 2nd longitudinal resonance at 14.87 kHz as a function of gas flow, it was found out that the peak amplitude of the PA signal increases. This is probably due to the fact that NO_2_ is a polar molecule with a tendency of being adsorbed onto the tubing and walls of the PA cell. The PA signal remains nearly constant above 1.5 standard liters per minute (SLPM). However, the signal-to-noise ratio (SNR) started to decrease above 2 SLPM because the acoustic background noise, due to a turbulent gas flow, was increased. At the resonance frequency of the fundamental mode the flow noise is more dominating. Therefore, only 1 SLPM sample gas flow was used in measurements that are presented in Figure 3. The estimated NO_2_ concentration in the MPAC was 20 ppm.

**Figure 4.**
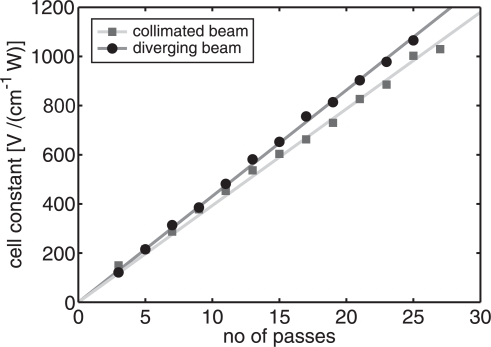
Cell constant of the PA cell as a function of laser beam passes. The total absorption of light by the NO_2_ molecules was approximately 0.01 in each measurement. At high numbers of passes the horizontally diverging laser beam enhances the sensitivity of the planar MPAC, when compared to the response achieved with the collimated beam, due to the better directionality of the generated pressure wave.

The cell constant of the MPAC is shown in Figure 4 as a function of number of laser beam passes with two different laser beam profiles. The collimated beam had a diameter of 0.8 mm. The horizontal width of the diverging beam was 0.2 mm at the entrance mirror of the MPAC, after which it diverts with a divergence angle of 0.6 mrad. At high number of laser beam passes the cell constant of the MPAC is about 10% larger with the diverging laser beam than with the collimated beam.

After 25 passes, for example, the width of the diverging laser beam was about 1.8 mm, whereas the width of the collimated beam remained at 0.8 mm. The beam spacing in the center of the planar PA resonator was about 1 mm with the 25 passes. Thus, the horizontally diverging beams overlap better with each other and generate an acoustic wave with a higher degree of directionality than with the spherical beams. A dense group of a large number of overlapping laser beams generates a uniform source for acoustic plane waves with negligible side lobes in the directionality pattern of the emitted sound. Instead, it is expected that a sparse group of separated beams produces a sound wave with prominent side lobes and a lower degree of directionality.

As the laser beams traverse diagonally as separate beams between the mirrors of MPAC with a low number of passes, it would be expected that the curves in Figure 4 showed a nonlinear dependence on the number of passes because of the changing directionality pattern. However, at the low number of passes, the reduced directionality of the generated sound wave is compensated by the increased, diagonal absorption path. Therefore, the measured dependencies of the cell constants in Figure 4 are fairly linear with both beam profiles.

By tilting the entrance mirror of the MPAC horizontally in such a way that the laser beam turns back between the mirrors, the number of beam passes is increased by more than a factor of two. About 70 passes were achieved, corresponding to cell constants of 3,120 VcmW^−1^ and 2,740 VcmW^−1^ with the diverging and the collimated beam, respectively.

The dielectric mirrors, used in the MPAC, had a 1-mm-wide, uncoated circular strip at the outer circumferences. The non-reflective areas limit the minimum spacing of the laser beams and, also, the maximum number of laser beam passes which can be achieved. On the other hand, the size and the divergence of the laser beam are also limiting factors. For example, with too high a divergence the light energy leaks easily from between the mirrors, and the PA signal enhancement is poor. The optimum beam divergence was not sought in this study. Instead, it was simply demonstrated that a horizontally diverging laser beam is a better choice for generating an acoustic plane wave than a collimated beam.

**Figure 5.**
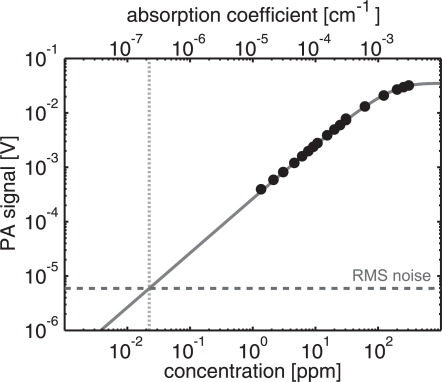
Photoacoustic signal as a function of NO_2_ concentration at the resonance frequency of the 2nd longitudinal mode. Central excitation with a diverging laser beam and about 70 passes was used. A simple curve, describing the Beer–Lambert’s law, was fitted to the measured data. The noise level is 5.9 *μ*V with 2.1 s measurement time, yielding a detection limit of 22 ppb for NO_2_ in N_2_.

The PA signal as a function of NO_2_ concentration is presented in Figure 5. The horizontally diverging laser beam was used, with the maximum number of 70 passes. Thus, the total absorption path length was 7 m inside the 10-cm-long PA resonator. Lower concentrations than 1 ppm were not diluted because of the nonlinear behavior of the mass flow controllers. The controllers had a linear dilution range approximately between 10%–100%. A curve of type 1 − exp(−*αL*), where *α* and *L* are the absorption coefficient and the total absorption path length, respectively, was fitted to the data of Figure 5. The equation describes the Beer–Lambert’s law for total absorption and does not take into account for the reflection losses of the mirrors. At normal incidence the reflectivity of the dielectric mirrors was 99.8%. The curve seems to fit the PA signal data well even at high absorption levels. For example, the optical density at 30 ppm is high, about 0.21, and the decrease from the linear approximation of Beer–Lambert’s law is about 10%. With a measurement time of 2.1 s the detection limit (SNR = 1) of NO_2_, diluted in N_2_ in NTP (normal temperature and pressure) conditions, is extrapolated to 22 ppb. This corresponds to a minimum detectable absorption coefficient and NNEA value of 2.2 × 10^−7^ cm^−1^ and 3.2 × 10^−9^ cm^−1^WHz^−1/2^, respectively.

Our results are summarized and compared with the results of other single and multipass PA methods in Table 1. In the case that only the minimum detectable absorption coefficient or concentration was given in the reference literature, a calculated NNEA value is indicated in Table 1, or vice versa. Furthermore, only studies where continuous-wave (CW) or quasi-CW light sources were used, are referenced in Table 1 because the NNEA value cannot be defined for a PA cell where single-pulse excitation of the PA signal is used. In quasi-CW mode of operation the PA signal is excited with a train of short pulses whose repetition rate is matched to the resonance frequency of the PA cell. Optimally, the quasi-CW excitation enhances the PA signal by a factor of *π*/2 compared to the CW excitation with the same average optical power [22]. The NNEA value of 3.2 × 10^−9^ cm^−1^WHz^−1/2^, achieved in this study, is close to smallest values that have been achieved with other resonant PA cells.

In future, the sensitivity of the PA detector where EMFIT films are used as a microphone, can be improved by increasing the number of stacked film layers and by replacing the self-made amplifier with another of lower electrical noise. In addition, the PA signal could be enhanced by using EMFIT films with higher sensitivity and by matching the electromechanical resonance of the stacked EMFIT films to the acoustic resonance of the PA cell. In order to reduce the electromechanical resonance frequency to a lower frequency than the 45 kHz, more than 5 layers need to be stacked. At a lower resonance frequency more efficient overlap between the laser beams and the standing pressure wave could also be achieved.

## 4. Conclusions

In this work, five stacked electromechanical films were applied as a transducer in a novel photoacoustic cell where both longitudinal acoustic resonances and optical multipasses were employed. The PA resonator was a simple rectangular structure with two steel plates, facing each other, and with open sides. The acoustic wave, resonating between the plates, was excited transversally in an optical multipass configuration. The laser beam, having a wavelength of 473 nm, was reflected between two planar dielectric mirrors so that the beams lay in a plane, parallel to the resonator plates of the PA cell.

A detection limit of 22 ppb for NO_2_ in N_2_ was achieved with the maximum number of 70 laser beam passes inside the PA cell and by using a measurement time and an average optical power of 2.1 s and 10 mW, respectively. The minimum detectable absorption and the normalized noise-equivalent absorption coefficients were 2.2 × 10^−7^ cm^−1^ and 3.2 × 10^−9^ cm^−1^WHz^−1/2^, respectively.

A sample gas flow of up to 2 liters per minute was used without a significant increase of the acoustic background noise at the second longitudinal resonance of 14.87 kHz where the acoustic quality factor was 175. Furthermore, it was demonstrated that the novel PA cell gives a comparable PA signal with a single laser beam pass than a corresponding cylindrical PA cell that are commonly used. The PA signal is easily enhanced by using multiple laser beam passes inside the planar PA cell.

The achieved sensitivity is close to the best values, measured from flowing gas samples at normal pressure. The sensitivity of the multipass PA cell, where electromechanical films are used as a microphone, can be further enhanced by optimizing the number of layered films, by using a low-noise electronic preamplifier, and by exploiting the electromechanical resonance of the microphone.

## Figures and Tables

**Table 1.** Comparison of PA methods with different number of optical passes in PA cell. *P*_laser_, *t*_m_, *α*_min_, and NNEA are the average laser power, measurement time, minimum detectable absorption coefficient, and normalized noise-equivalent absorption, respectively.

	Parameters				
Study	No of passes	*P*_laser_ [mW]	*t*_m_ [s]	*α*_min_* [10^−8^cm^−1^]	NNEA^*^ [10^−9^cm^−1^WHz^−1/2^]
This	70	10	2.1	22	3.2
[18]	10	126	0.3	2.6	1.8^†^
[12]	36	6, 000	30	0.069	22.7^†^
[23]	2	500	10	0.95	1.5
[24]	1	6.6	1	40^†^	2.6
[25]	1	30	2.6	0.36^‡^	0.17^‡^

* For SNR = 1

† A calculated value

‡ For nonflowing gas sample
